# Role of Reactive Oxygen Species in the Abrogation of Oxaliplatin Activity by Cetuximab in Colorectal Cancer

**DOI:** 10.1093/jnci/djv394

**Published:** 2015-12-29

**Authors:** Valeria Santoro, Ruochen Jia, Hannah Thompson, Anke Nijhuis, Rosemary Jeffery, Konstantinos Kiakos, Andrew R. Silver, John A. Hartley, Daniel Hochhauser

**Affiliations:** **Affiliations of authors:**Cancer Research UK Drug-DNA Interactions Research Group, UCL Cancer Institute, University College London, London, UK (VS, RJ, KK, JAH, DH); Colorectal Cancer Genetics Group, Blizard Institute, London, UK (HT, AN, RJ, ARS);

## Abstract

**Background:**

The antibody cetuximab, targeting epidermal growth factor receptor (EGFR), is used to treat metastatic colorectal cancer (mCRC). Clinical trials suggest reduced benefit from the combination of cetuximab with oxaliplatin. The aim of this study was to investigate potential negative interactions between cetuximab and oxaliplatin.

**Methods:**

Thiazolyl blue tetrazolium bromide (MTT) assay and Calcusyn software were used to characterize drug interactions. Reactive oxygen species (ROS) were measured by flow cytometry and real-time polymerase chain reaction oxidative stress arrays identified genes regulating ROS production. Chromatin immunoprecipitation (ChIP) measured signal transducer and activator of transcription 1 (STAT-1) binding to dual oxidase 2 (DUOX2) promoter. SW48, DLD-1 KRAS wild-type cell lines and DLD-1 xenograft models exposed to cetuximab, oxaliplatin, or oxaliplatin + cetuximab (control [saline]; n = 3 mice per treatment group) were used. Statistical tests were two-sided.

**Results:**

Cetuximab and oxaliplatin exhibited antagonistic effects on cellular proliferation and apoptosis (caspase 3/7 activity reduced by 1.4-fold, 95% confidence interval [CI] = 0.78 to 2.11, *P* = .003) as opposed to synergistic effects observed with the irinotecan metabolite 7-Ethyl-10-hydroxycamptothecin (SN-38). Although both oxaliplatin and SN-38 produced ROS, only oxaliplatin-mediated apoptosis was ROS dependent. Production of ROS by oxaliplatin was secondary to STAT1-mediated transcriptional upregulation of DUOX2 (3.1-fold, 95% CI = 1.75 to 2.41, *P* < .001). Inhibition of DUOX2 induction and p38 activation by cetuximab reduced oxaliplatin cytotoxicity.

**Conclusions:**

Inhibition of STAT1 and DUOX2-mediated ROS generation by cetuximab impairs p38-dependent apoptosis by oxaliplatin in preclinical models and may contribute to reduced efficacy in clinical settings. Understanding the rationale for unexpected trial results will inform improved rationales for combining EGFR inhibitors with chemotherapeutic agents in future therapeutic use.

In view of the importance of the epidermal growth factor receptor (EGFR) in the development and maintenance of human cancers, there is considerable interest in inhibiting this pathway with monoclonal antibodies or small molecule inhibitors (1–4). Antibodies inhibiting EGFR, including cetuximab and panitumumab, have shown efficacy in colorectal cancer (CRC) either as monotherapy, or in combination with chemotherapy (5–8).

Preclinical and clinical studies of cetuximab or panitumumab with irinotecan-based chemotherapy have shown benefit in CRC (9–10). In contrast, despite some efficacy for antibodies targeting EGFR and oxaliplatin combinations (11–12), other studies have suggested either no benefit or a negative interaction. A randomized study using cetuximab in combination with oxaliplatin and fluoropyrimidines to treat CRC showed no benefit from addition of cetuximab (13). More recently, the randomized NEW EPOC study of oxaliplatin and 5-fluorouracil alone or combined with cetuximab demonstrated reduced progression-free and overall survival with cetuximab (14).

Cisplatin and oxaliplatin induce intra- and interstrand DNA cross-links, DNA-protein adducts (15–17), and generate formation of reactive oxygen species (ROS) and toxic oxygen metabolites, which cause cytotoxic effects by inducing DNA damage and apoptosis (18–21). Given lack of synergy and possible antagonism of oxaliplatin combined with cetuximab in CRC, we investigated potential mechanisms of interaction.

## Methods

### Reagents and Antibodies

Cetuximab (5mg/mL) was obtained from Merck Serono KGaA (Darmstadt, Germany). EMD Serono (Boston, MA) provided the MEK inhibitor pimasertib. SN-38, p38 inhibitor (SB202190), N-Acetyl-L-Cysteine (NAC), L-Ascorbic acid, and MTT were purchased from Sigma-Aldrich. Reagents/antibodies used for immunoblotting are listed in the Supplementary Methods (available online).

### Cell Lines and Culture Conditions

Merck Serono (Darmstadt) provided the SW48 cell line, and Bert Vogelstein (Johns Hopkins University) the DLD-1 isogenic KRAS wild-type cell line. Cells were cultured in McCoy’s 5A modified media (Sigma-Aldrich), supplemented with 10% fetal bovine serum (Gibco), 2mM L-glutamine (Sigma), and 2mM penicillin-streptomycin (PAA). Cell lines were authenticated in May 2015 (LGC standards).

### Immunoblotting

Immunoblotting was performed as described (22). Detailed methods are provided in the Supplementary Materials (available online).

### ROS Detection

ROS levels were detected with the cell-permeable compound H_2_DCFDA (Life Technologies). Drug-treated cells or control cells were washed twice in PBS and then incubated with PBS-H_2_DCFDA at 37° (1 μM) for 30 minutes. Following incubation with the ROS indicator, cells were washed twice in PBS, trypsinized and collected. Samples were analyzed using a flow cytometer (CyAn ADP), and ROS was measured as mean fluorescence intensity. Results were analyzed with the Summit v4.3 software.

### Apoptosis and Cell Viability Measurement

Apoptosis was measured by Caspase 3/7 Glo assay and cell viability by Cell Titre Glo assay (Promega) according to the manufacturer’s protocol.

### Drug Combination Assays

Ten thousand cells per well were seeded in a 96-well plate (Corning) and drug-treated for 72 hours with cetuximab, oxaliplatin, SN-38, or their combination; inhibition of proliferation was measured by MTT assay. Synergy or antagonism were determined with Calcusyn software using methodology of Chou and Talalay (23). Drug scheduling and dosing is provided in the Supplementary Materials (available online).

### Real-Time Polymerase Chain Reaction Oxidative Stress Arrays

Real-time polymerase chain reaction (RT-PCR) oxidative stress arrays (Qiagen) were used to measure RNA expression of stress-related genes following cetuximab and oxaliplatin treatment. Additional details are provided in the Supplementary Materials (available online).

### Chromatin Immunoprecipitation

SW48 cells were treated with oxaliplatin (50 μM), and proteins were cross-linked with 1% formaldehyde (Sigma-Aldrich) for 10 minutes at ambient temperature. Cells were lysed and chromatin extracted, and STAT-1 binding to the DUOX2 promoter was assessed by RT-PCR. Detailed assay protocol is described in the Supplementary Materials (available online).

### Xenografts

All in vivo experiments were performed according to the Animal Research Ethics and United Kingdom Coordination Committee on Cancer Research Guidelines and Home Office Regulations (project license PPL70/7411). Mice were housed under specific pathogen-free conditions and all procedures involving mice conducted according to the requirements of the United Kingdom Home Office Animals (Scientific Procedures) Acts, 1986. DLD-1 cells (5 x 10^6^ in 200 µL saline) were injected subcutaneously into a site in the flank of each female nude mouse (nu/nu) around eight weeks of age. Xenografts attained a predetermined size (200–300mm diameter), and then mice were assigned randomly to one of four experimental groups: control and n = 3 for each treatment group (cetuximab, oxaliplatin, oxaliplatin + cetuximab) and were treated appropriately. Drugs were administered by intraperitoneal injection as follows: control (saline), cetuximab (30mg/kg in saline), oxaliplatin (8mg/kg in saline), oxaliplatin + cetuximab (as before). After 24 hours, mice were killed and xenografts were harvested. Tumors were processed to measure DUOX2 and DUOXA2 mRNA levels by RT-PCR and in situ hybridization (ISH); DUOX2, Ki67, and cleaved caspase 3 were measured by immunohistochemistry (IHC). Additional experimental procedures are provided in the Supplementary Materials (available online).

### Statistical Analysis

Data was analyzed using the Student’s *t* test or two-way analysis of variance (ANOVA) as appropriate for analysis. Unpaired and Student’s *t* tests were used when comparing two treatment groups. Welch’s correction was applied where appropriate. For analysis of more than two groups, two-way ANOVA with multiple comparisons correction was applied (Bonferroni post-tests). Results were considered statistically significant at a *P* value of less than .05. Means, standard deviations, 95% confidence intervals (CIs), and statistical significance were calculated using GraphPad Prism (software version 6.0d). All statistical tests were two-sided.

## Results

### Cellular Effects of Cetuximab and Oxaliplatin Combinations In Vitro

**Figure 1.**
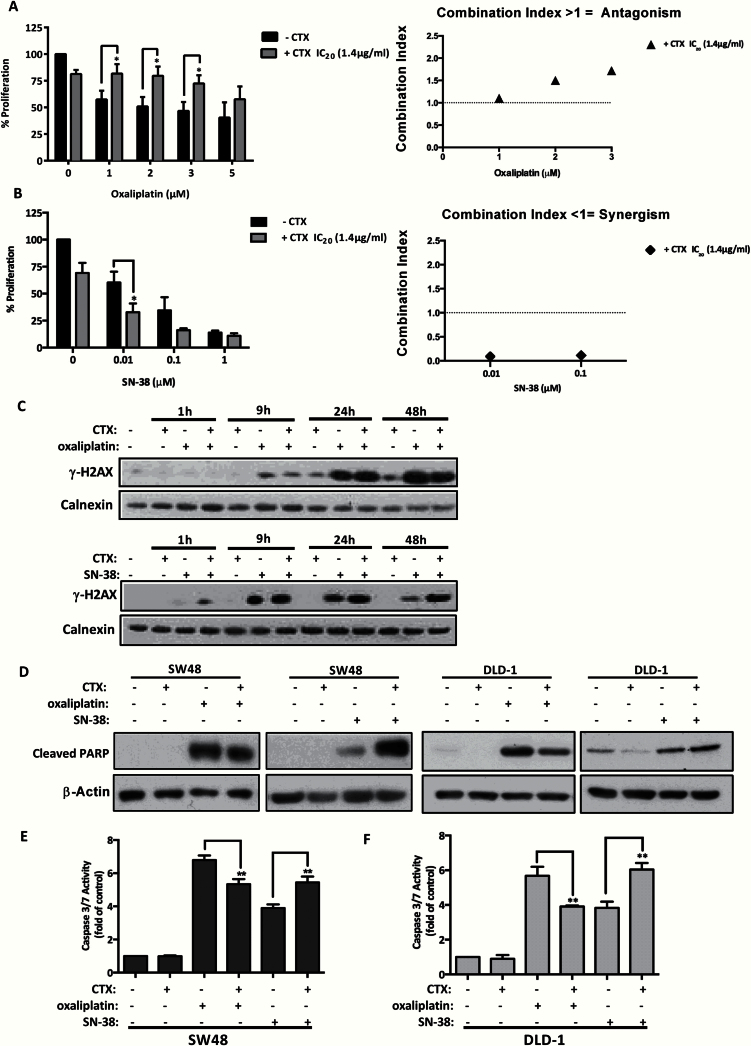
Combination of cetuximab and oxaliplatin in vitro. **A and B**) Thiazolyl blue tetrazolium bromide (MTT) assay assessed the effect of a fixed concentration of cetuximab (IC_20_ 1.4 μg/mL) with oxaliplatin (1-2-3-5 μM) or SN-38 (0.01–1 μM) on growth inhibition at 72 hours in SW48 cells. Results are presented as mean ± SD of three independent experiments. Statistical significance was calculated by two-tailed Student’s *t* test (*P < .05, †*P* < .01, and ‡*P* < .001). Combination index calculations were performed by Calcusyn software. Values of 1 indicate additivity; values greater than 1 indicate antagonism and of less than 1 indicate synergism. **C**) H2AX phosphorylation was detected following continuous treatment of 50 μM oxaliplatin (3 hours) or 1 μM SN-38 (1 hour) in combination with 100 μg/mL cetuximab in SW48 cells. Protein samples were collected post-treatment at the indicated times, and calnexin was used as a loading control. The result of three independent experiments is presented. **D**) SW48 and DLD-1 cells were treated with oxaliplatin (50 μM) or SN-38 (1 μM) and cetuximab (100 μg/mL) and with oxaliplatin (100 μM) or SN-38 (1 μM) and cetuximab (100 μg/mL) for 18 hours, respectively. β-Actin was used as loading control. The result of three independent experiments is presented. **E and F**) Apoptosis was measured by caspase 3/7 activity. SW48 cells were treated with oxaliplatin (50 μM), SN-38 (1 μM), or their combination with cetuximab (100 μg/mL) for 18 hours. Isotoxic concentrations (IC_75_) of oxaliplatin (100 μM) and SN-38 (1 μM) were used for the DLD-1 cells. Results are presented as fold-increase to untreated samples and as mean ± SD of three independent experiments. Statistical significance was calculated by two-tailed Student’s *t* test (*P < .05, †*P* < .01, and ‡*P* < .001).

Cetuximab induced dose-dependent inhibition of EGFR, AKT, and ERK activation in SW48 and DLD-1 KRAS wild-type cells (Supplementary Figure 1, available online). The combination of cetuximab (IC_20_ dose = 1.4 μg/mL) with 1–3 μM oxaliplatin statistically significantly increased cellular proliferation by 24.3% (95% CI = 4.98 to 43.64, *P =* .02), by 28.7% (95% CI = 8.98 to 48.54, *P =* .01), and by 25.6% (95% CI = 7.44 to 43.93, *P =* .01), respectively, as compared with cells treated with oxaliplatin alone (Figure 1A). In contrast, addition of cetuximab to 0.01 μM SN-38 reduced cellular growth by 27.5% (95% CI = 6.87 to 48.43, *P =* .02) and by 18.3% (95% CI = -1.52 to 38.11, *P* = .06) at 0.1 μM SN-38 (Figure 1B). Combination indices describing the interaction of oxaliplatin with cetuximab are greater than 1 and indicate antagonism whereas combination indices obtained for the combination of cetuximab with SN-38 are less than 1 and indicate synergism (Figure 1A-B).

The effects of cetuximab treatment on DNA damage and apoptosis were measured. Addition of cetuximab to oxaliplatin had no effect on H2AX phosphorylation at all time points compared with oxaliplatin alone (Figure 1C). Immunoblotting demonstrated that levels of PARP cleavage induced by oxaliplatin were reduced by cetuximab in both SW48 and DLD-1 cells (Figure 1D). In support, cetuximab statistically significantly reduced the levels of caspase 3/7 enzyme activity produced by oxaliplatin from 6.7- to 5.3-fold (95% CI = 0.78 to 2.11, *P =* .003) in the SW48 cells (Figure 1E) and from 5.6- to 3.9-fold (95% CI = 0.92 to 2.60, *P* = .004) in the DLD-1 KRAS wild-type cell line (Figure 1F).

However, when cetuximab was added to SN-38, increased induction of γ-H2AX and cleaved PARP levels were observed (Figure 1, C and D). Accordingly, caspase 3/7 activity induced by SN-38 increased 1.5-fold (95% CI = 0.87 to 2.21, *P =* .003) in the presence of cetuximab in SW48 cells and 2.2-fold (95% CI = 1.36 to 3.05, *P =* .001) in DLD-1 KRAS wild-type cells (Figure 1, E and F).

### Production of ROS by Oxaliplatin and SN-38 in Colorectal Cancer Cells

**Figure 2.**
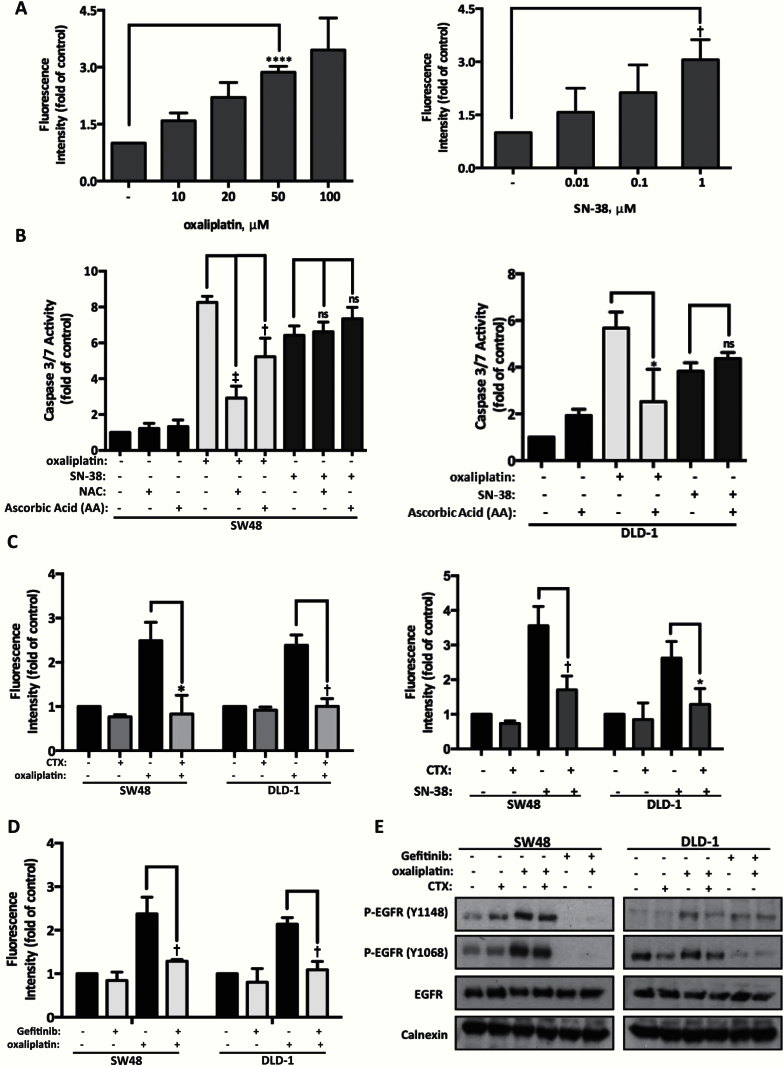
Cetuximab and oxaliplatin treatment effects on cellular reactive oxygen species (ROS) levels. **A**) SW48 cells were treated with oxaliplatin (10–100 μM) or SN-38 (0.01–1 μM) for one hour. ROS were detected with H_2_DCFDA reagent and flow cytometry analysis. Results are presented as fold-increase in mean fluorescence intensity normalized to untreated and mean ± SD (n = 3). **Symbols** indicate statistical significance (two-tailed Student’s *t* test; *P < .05, †*P* < .01, and ‡*P* < .001). **B**) Caspase 3/7 activity detected apoptosis following 24 hours’ continuous treatment of oxaliplatin (50 μM), SN38 (1 μM), or their combination with the antioxidants NAC (1mM) or ascorbic acid (100 μM) in the SW48 cell line and oxaliplatin (100 μM) or SN-38 (1 µM) with ascorbic acid (100 µM) in DLD-1 cells. Results are presented as fold-increase to untreated sample and are shown as mean ± SD (n = 3). Statistical analysis conducted as above. **C and D**) Levels of ROS were measured as described in **(A)**. SW48 and DLD-1 cells were treated with 50 μM or 100 μM oxaliplatin, respectively, SN-38 (1 µM), cetuximab (100 μg/mL), or gefitinib (1 µM) for one hour. Results are presented as fold-increase in mean fluorescence intensity normalized to untreated control. Each experiment was repeated three times, and results are shown as mean ± SD. **Symbols** indicate statistical significance (*P < .05, †*P* < .01, and ‡*P* < .001), and two-tailed Student’s *t* test was used for analysis. **E**) SW48 and DLD-1 cells were treated as in **(C and D)**, and samples processed by immunoblotting calnexin were used as a loading control. The result of three independent experiments is presented.

Exposure to 50 μM oxaliplatin statistically significantly increased ROS levels by 1.8-fold (oxaliplatin vs untreated, 95% CI = 1.62 to 2.17, *P* < .001) (Figure 2A), and, similarly, treatment with 1 μM SN-38 elevated ROS production by 2.0-fold (SN-38 vs untreated, 95% CI = 1.15 to 2.96, *P =* .003) (Figure 2A).

Levels of caspase 3/7 activity induced by oxaliplatin were statistically significantly reduced from 8.2- to 2.9-fold (95% CI = 4.15 to 6.54, *P <* .001) upon addition of the antioxidant N-acetyl cysteine (NAC) and to 5.2-fold (95% CI = 1.29 to 4.77, *P =* .008) in the presence of ascorbic acid (AA) in SW48 cells (Figure 2B). Apoptosis induced by oxaliplatin was also reduced from 5.6- to 2.5-fold (95% CI = 0.67 to 5.63, *P =* .02) in combination with AA in DLD-1 cells (Figure 2B). In contrast, SN-38-induced apoptosis was not affected by antioxidant treatment in both cell lines tested (Figure 2B). NAC and AA are capable of reducing levels of ROS (H_2_O_2_ treatment) from 6.1-fold to 1.1-fold (95% CI = 2.72 to 7.28, *P =* .003) and to 1.9-fold (95% CI = 1.91 to 6.54, *P =* .007), respectively, in SW48 cells (Supplementary Figure 2A, available online).

We assessed effects of cetuximab treatment on ROS production when combined with oxaliplatin and SN-38. ROS levels produced by oxaliplatin were statistically significantly reduced from 2.4- to 0.8-fold (95% CI = 0.30 to 2.99, *P =* .02) in the SW48 cells and from 2.3- to 1.0-fold (95% CI = 0.68 to 2.07, *P =* .005) in the DLD-1 cells following addition of cetuximab (Figure 2C). Cetuximab also statistically significantly reduced levels of SN-38-induced oxidative stress in both cell lines (Figure 2C). To probe if ROS reduction by cetuximab was mediated by EGFR inhibition, levels of ROS were measured following oxaliplatin and EGFR inhibitor gefitinib. A dose of gefitinib (1 μM), which blocked activation of the EGFR pathway in SW48 and DLD-1 cells, was used to assess effects on ROS production (Supplementary Figure 3, available online). Oxaliplatin-induced ROS were reduced from 2.3- to 1.2-fold (95% CI = 0.46 to 1.70, *P =* .008) in SW48 cells and from 2.1- to 1.0-fold (95% CI = 0.65 to 1.43, *P =* .002) in DLD-1 cells by gefitinib treatment (Figure 2D).

Oxaliplatin-induced EGFR phosphorylation was inhibited by cetuximab, gefitinib, and EGFR knockdown (Figure 2E; Supplementary Figure 2B, available online). Similar to the effect observed with cetuximab or gefitinib, ROS produced by oxaliplatin was statistically significantly reduced from 2.2- to 0.9-fold (scrambled-oxaliplatin vs EGFR siRNA-oxaliplatin, 95% CI = 0.20 to 2.31, *P =* .02) upon EGFR knockdown (Supplementary Figure 2B, available online).

### Effects of Cetuximab on ROS Production by Oxaliplatin

**Figure 3.**
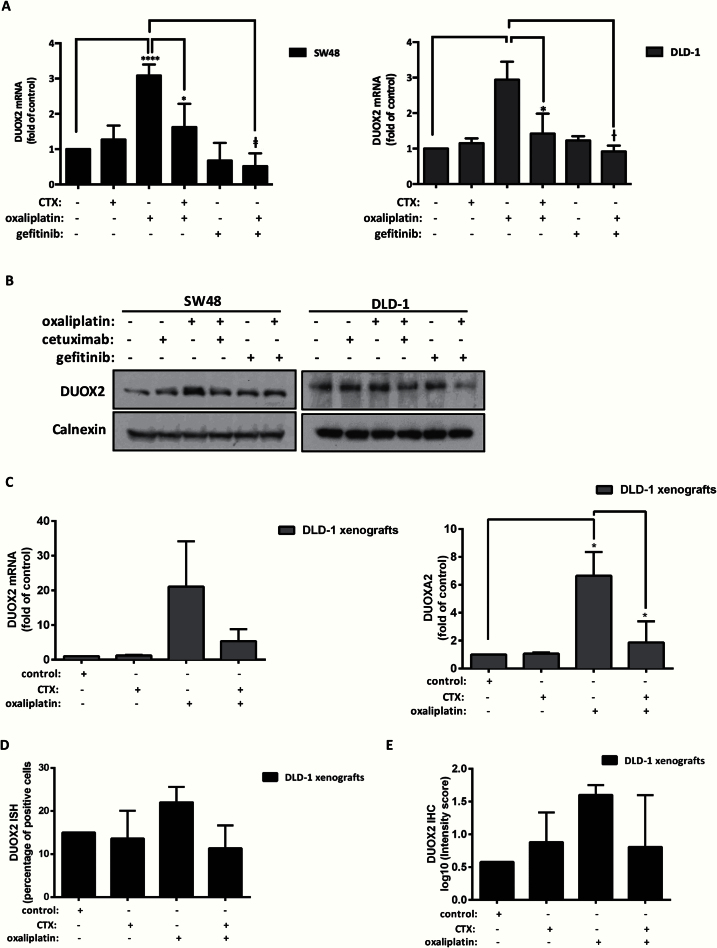

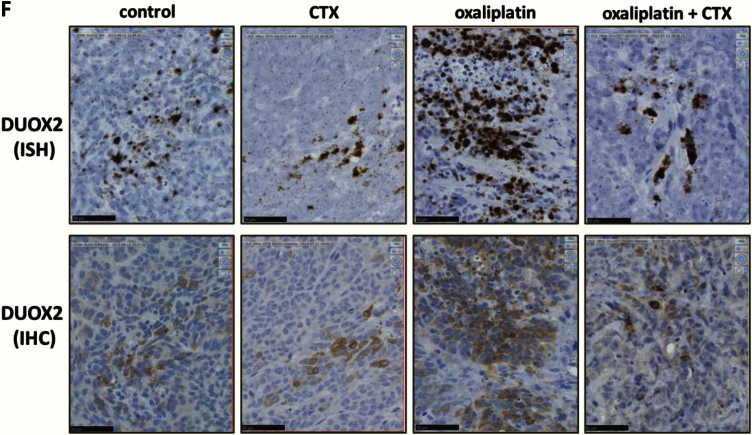
In vitro and in vivo expression of DUOX2 following oxaliplatin and cetuximab treatment. **A**) SW48 cells were treated with cetuximab (100 μg/mL), gefitinib (1 μM), oxaliplatin (50 μM), or their combination for six hours. DLD-1 cells were treated with cetuximab (100 μg/mL), gefitinib (1 μM), and oxaliplatin (100 μM). mRNA levels of DUOX2 were measured by real-time polymerase chain reaction (RT-PCR). Results (2^-ΔΔCT^) are presented as mean ± SD of three independent experiments. GADPH was used as endogenous control and untreated sample as calibrator. Statistical significance was calculated by two-tailed Student’s *t* test (*P < .05, †*P* < .01, and ‡*P* < .001). **B**) SW48 and DLD-1 cells were treated in the same conditions described in **(A)** and sample processed by immunoblotting. Calnexin was used as a loading control. The result of three independent experiments is presented. **C**) 5 x 10^6^ DLD-1 cells were injected into a single site on the flank of nude mice (nu/nu). Mice were assigned randomly to one of four experimental groups: control and n = 3 for each treatment group and drug treated for 24 hours by intraperitoneal injection with control (saline), cetuximab (30mg/kg in saline), oxaliplatin (8mg/kg in saline), oxaliplatin + cetuximab (as before). mRNA for DUOX2 **(C, left panel)** and DUOXA2 **(C, right panel)** was determined using real-time polymerase chain reaction and results expressed as a fold-change (mean ± SD) normalized to untreated control. Xenograft serial sections (5 μM) were prepared and DUOX2 in situ hybridization (ISH) **(D)**, and DUOX2 immunohistochemistry (IHC) **(E)** was conducted. **F**) Representative images at 40x magnification resulting from ISH and IHC analysis (**scale bars** = 50 µM). Results are expressed as mean ± SD. Xenograft data was normally distributed according to Shapiro-Wilks testing, except IHC, where data was log_10_-transformed and then determined to be normally distributed. Statistics was performed using two-tailed Student’s *t* test with Welch’s correction.

The mRNA expression of 84 genes involved in the oxidative stress pathway was assessed by RT-PCR oxidative stress array. Genes whose expression was altered at least two-fold following treatment with oxaliplatin or combination with cetuximab in SW48 cells are given in Table 1. mRNA expression of DUOX2, a member of the NADPH oxidase ROS-generating enzyme family, was statistically significantly upregulated by oxaliplatin but reduced in combination treatment (Table 1). Validation of these results by RT-PCR showed DUOX2 mRNA was increased upon oxaliplatin treatment by 3.1-fold (oxaliplatin vs untreated, 95% CI = 1.75 to 2.41, *P <* .001) in SW48 cells and 2.9-fold (95% CI = 1.13 to 2.75, *P =* .002) in DLD-1 cells (Figure 3A). Cetuximab reduced DUOX2 mRNA levels from 3.1- to 1.6-fold (oxaliplatin vs oxaliplatin + CTX, 95% CI = 0.28 to 2.63, *P =* .02) and gefitinib from 3.0- to 0.5-fold (oxaliplatin vs oxaliplatin + gefitinib, 95% CI = 1.79 to 3.33, *P <* .001) in the SW48 cells (Figure 3A). Similarly, cetuximab and gefitinib inhibited oxaliplatin-induced DUOX2 mRNA upregulation by 1.5-fold (oxaliplatin vs oxaliplatin + CTX, 95% CI = 0.3 to 2.7, *P =* .02) and by 2.0-fold, respectively (oxaliplatin vs oxaliplatin + gefitinib, 95% CI = 1.21 to 2.87, *P =* .003) in DLD-1 cells (Figure 3A).

**Table 1.** Differentially expressed genes in oxaliplatin or oxaliplatin and cetuximab treatment groups as assessed by RT-PCR oxidative stress array analysis*

Gene symbol	Refseq	Oxaliplatin Fold-change (95% CI)	Oxaliplatin *P*	Oxaliplatin and cetuximab Fold-change (95% CI)	Oxaliplatin and cetuximab *P*
AOX1	NM_001159	2.0 (0.78 to 3.26)	.05	2.3 (0.38 to 4.13)	.12
CCL5	NM_002985	4.0 (2.17 to 5.86)	.002	3.0 (1.00 to 5.00)	.09
NCF1	NM_000265	5.0 (0.00 to 10.32)	.02	1.7 (0.69 to 2.74)	.16
DUOX2	NM_014080	2.8 (1.88 to 3.90)	<.001	1.7 (0.45 to 3.00)	.26
DUSP1	NM_004417	2.1 (1.30 to 2.92)	.006	2.1 (1.58 to 2.69)	.004
GPX7	NM_015696	2.3 (0.00 to 7.59)	.14	1.0 (0.00 to 3.40)	.56
GSTZ1	NM_001513	-2.1 (0.15 to 0.82)	.25	-1.7 (0.00 to 1.19)	.36
GTF2I	NM_001518	-2.3 (0.19 to 0.67)	.05	-2.2 (0.29 to 0.63)	.03
MSRA	NM_012331	-2.3 (0.21 to 0.63)	.06	-2.4 (0.12 to 0.69)	.05
NCF2	NM_000433	-2.3 (0.09 to 0.75)	.16	-2.2 (0.07 to 0.81)	.12
OXR1	NM_181354	-2.6 (0.10 to 0.66)	.12	-2.2 (0.15 to 0.73)	.13
IPCEF1	NM_015553	-2.4 (0.00 to 0.84)	.20	-3.4 (0.00 to 0.59)	.09
PRDX5	NM_181652	-2.4 (0.06 to 0.76)	.14	-2.0 (0.14 to 0.86)	.16
DHCR24	NM_014762	-2.2 (0.18 to 0.74)	.19	-3.2 (0.01 to 0.61)	.14
PXDNL	NM_144651	-2.0 (0.19 to 0.81)	.16	-1.6 (0.24 to 0.94)	.19

* Fold-change, 95% confidence interval, and *P* value obtained from two-sided Student’s *t* test are shown for oxaliplatin or oxaliplatin and cetuximab treatment groups when compared with the untreated sample in the SW48 cells. CI = confidence interval; RT-PCR = real-time polymerase chain reaction.

Protein expression of DUOX2 was also increased by oxaliplatin in SW48 and DLD-1 cells (Figure 3B). DUOX2 upregulation persisted up to 24 hours following oxaliplatin treatment (data not shown). Conversely, cetuximab and gefitinib treatment reduced DUOX2 protein induction by oxaliplatin (Figure 3B). Densitometry analysis indicates a reduction from 3.4- to 1.1-fold (oxaliplatin vs oxaliplatin + CTX, 95% CI = 0.75 to 3.84, *P =* .01) in SW48 cells (Supplementary Figure 4A, available online) and from 2.6- to 1.2-fold in DLD-1 cells (oxaliplatin vs oxaliplatin + CTX, 95% CI = 0.25 to 2.60, *P =* .02) following cetuximab treatment (Supplementary Figure 4B, available online). Levels of dual oxidase maturation factor 2 (DUOXA2) involved in DUOX2 protein maturation and expression in active enzymatic form were also increased upon oxaliplatin treatment (Supplementary Figure 4, C and D, available online).

Oxaliplatin-induced ROS was measured following DUOX2 knockdown. siRNA-mediated targeting of DUOX2 statistically significantly reduced levels of ROS produced by oxaliplatin from 3.31- to 1.5-fold (scrambled-oxaliplatin vs DUOX2 siRNA-oxaliplatin, 95% CI = 0.00 to 3.61, *P =* .05) (Supplementary Figure 4E, available online).

To assess these changes in vivo, expression of DUOX2 and DUOXA2 was measured in DLD-1 cells within xenografts in nude mice 24 hours after treatment. DUOX2 mRNA levels relative to control were increased by oxaliplatin (21.1-fold increase, 95% CI = -12.30 to 52.52, *P =* .11) and reduced by combination with cetuximab to 5.3-fold (95% CI = -45.61 to 14.10, *P =* .16) (Figure 3C). Analysis of DUOXA2 revealed a pattern similar to DUOX2 results; oxaliplatin upregulated expression statistically significantly (6.6-fold increase, 95% CI = 1.39 to 9.88, *P =* .02), while oxaliplatin + CTX decreased DUOXA2 expression by 4.77-fold (95% CI = -8.45 to -1.10, *P =* .02) (Figure 3C).

Results from DUOX2 ISH and IHC performed on xenograft serial sections were in accord with RT-PCR findings. Oxaliplatin treatment increased DUOX2 mRNA and protein expression more than cetuximab or oxaliplatin + CTX although differences between treatments were not statistically significant (Figure 3, D and E). In Figure 3F, a representative image (40x magnification) of matched sections of DUOX2 ISH and IHC is presented; lower power images (20x magnification) are shown in Supplementary Figure 5 (available online).

Proliferation rates measured by Ki67 positivity were higher in oxaliplatin + CTX–treated xenografts (14.8% increase, 95% CI = 0.25 to 29.53, *P =* .04) (Supplementary Figure 6A, available online). Conversely, apoptosis by positivity for cleaved caspase 3 was highest in oxaliplatin-treated mice compared with cetuximab and combined drugs (Supplementary Figure 6B, available online).

### STAT1 Binding to DUOX2 Promoter in Response to Oxaliplatin

**Figure 4.**
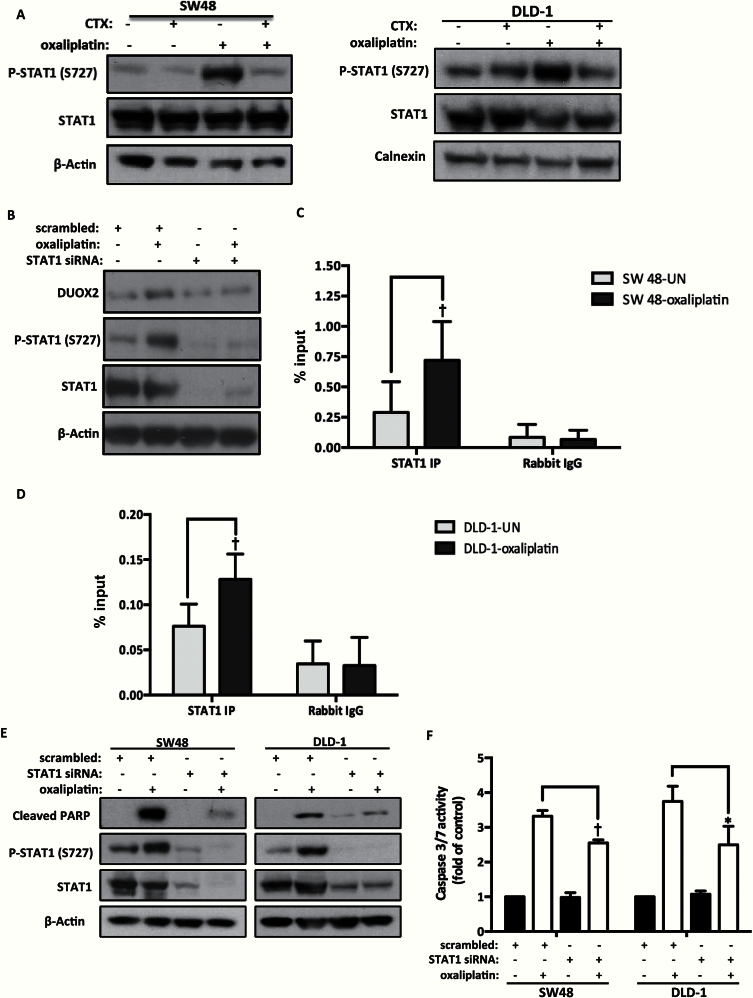
Regulation of DUOX2 transcription by oxaliplatin and cetuximab. **A**) SW48 and DLD-1 cells were treated with 50 μM or 100 μM oxaliplatin, respectively, in combination with cetuximab (100 μg/mL) for one hour. β-actin and calnexin were used as loading controls. The result of three independent experiments is presented. **B**) Following 72 hours’ transfection with scrambled siRNA or STAT1 siRNA (50nM), the SW48 cells were treated with oxaliplatin (50 μM) for six hours. β-actin was used as a loading control. The result of three independent experiments is presented. **C and D**) Following three hours’ oxaliplatin treatment (50 μM and 100 μM in SW48 and DLD-1 cells, respectively), binding of STAT1 to the DUOX2 promoter was measured by chromatin immunoprecipitation. Rabbit IgG was used as negative control. Values are normalized to INPUT samples and presented as % input. Each experiment was repeated in triplicates, and results are presented as mean ± SD. Statistical analysis was performed with two-way analysis of variance (ANOVA) and Bonferroni post-test. Correction for multiple comparisons was applied for STAT1 IP-oxaliplatin vs STAT1 IP-untreated and rabbit IgG-oxaliplatin vs rabbit Ig-untreated for both cell lines (*P < .05, †*P* < .01, and ‡*P* < .001). **E**) Following 72 hours’ transfection (50nM) with STAT1 siRNA, the SW48 and DLD-1 cells were treated with 50 μM or 100 μM oxaliplatin, respectively, for 18 hours. Levels of PARP cleavage measured apoptosis and β-Actin was used as a loading control. The experiment presented is representative of three independent experiments. **F**) Caspase 3/7 activity was measured in the same experimental conditions in **(E)**. Results are presented as mean ± SD of three independent experiments. Statistical analysis was performed with two-way ANOVA and Bonferroni post-test. Correction for multiple comparisons was applied for scrambled vs scrambled-oxaliplatin, scrambled vs STAT1 siRNA, scrambled vs scrambled-oxaliplatin, scrambled-oxaliplatin vs STAT1 siRNA, and scrambled-oxaliplatin vs STAT1 siRNA-oxaliplatin.

Our results show that oxaliplatin treatment increases STAT1 phosphorylation and that activation is inhibited by cetuximab in SW48 and DLD-1 cells (Figure 4A). Furthermore, siRNA-mediated inhibition of STAT1 expression prevented DUOX2 induction by oxaliplatin in SW48 cells (Figure 4B). Chromatin immunoprecipitation (ChIP) was performed to assess STAT1 binding to a specific sequence in the DUOX2 promoter (5’-TTCCTGCAA-3’), which differs from the canonical GAS (interferon-γ activated sequence) element by one nucleotide (24). Treatment of cells with oxaliplatin statistically significantly increased STAT1 binding to the DUOX2 promoter region in the SW48 cell line (STAT1 IP-oxaliplatin vs STAT1 IP-untreated, 95% CI = 0.23 to 0.62, *P =* .002) (Figure 4C) and in the DLD-1 cell line (STAT1 IP-oxaliplatin vs STAT1 IP-untreated, 95% CI = 0.03 to 0.07, *P =* .001) (Figure 4D).

Furthermore, levels of P-STAT1 and cleaved PARP produced by oxaliplatin were reduced by STAT1 knockdown in SW48 and DLD-1 cells (Figure 4E). Caspase 3/7 activity was also reduced from 3.3- to 2.5-fold (scrambled-oxaliplatin vs STAT1 siRNA-oxaliplatin, 95% CI = 0.08 to 1.46, *P =* .02) in SW48 cells and from 3.7- to 2.4-fold (scrambled-oxaliplatin vs STAT1 siRNA-oxaliplatin, 95% CI = 0.56 to 1.93, *P <* .001) in DLD-1 cells (Figure 4F).

### ROS-Dependent Inhibition of p38 Activity on Modulates Apoptosis by Oxaliplatin

**Figure 5.**
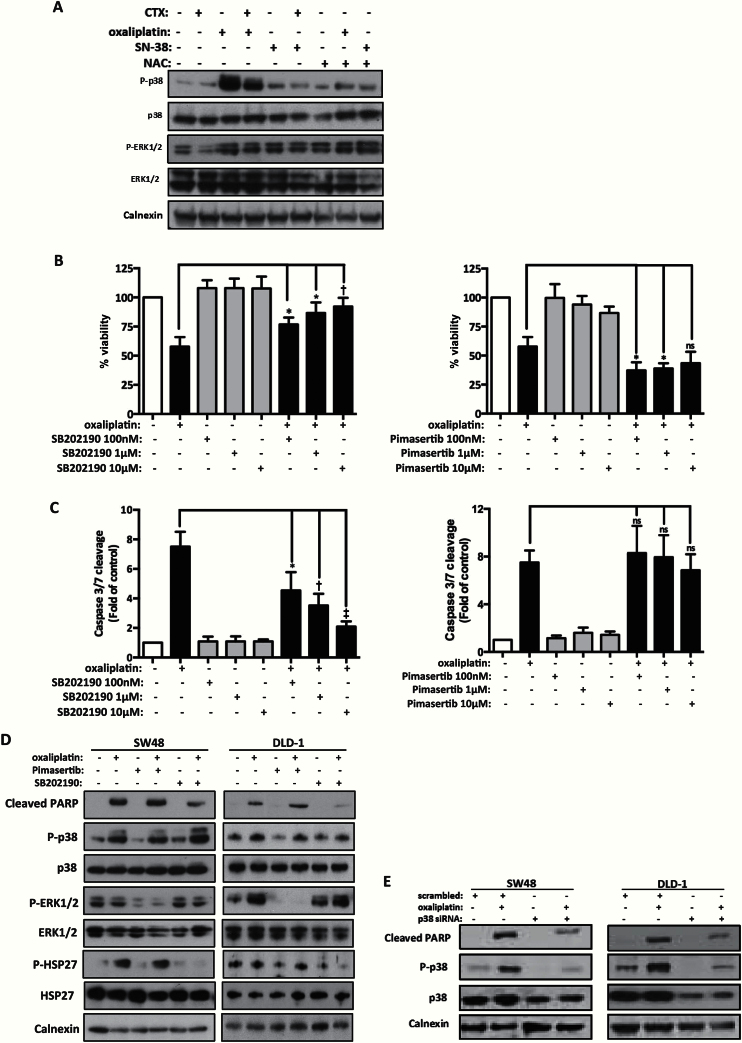
Role of p38 and ERK kinases in oxaliplatin-induced apoptosis. **A**) Activation of p38 and ERK1/2 was assessed by immunoblotting in the SW48 cells treated with oxaliplatin (50 μM), cetuximab (100 μg/mL), and the antioxidants NAC (1mM) or ascorbic acid (AA) (100 μM) for 24 hours. Calnexin was used as a loading control. The result of three independent experiments is presented. **B**) SW48 cells were treated with oxaliplatin (50 μM), SB202190 (0.1-1-10 μM), or pimasertib (0.1-1-10 μM) for 24 hours. Viable cells (%) were measured by the Cell Titre Glo assay (Promega), and treated samples were normalized to untreated control (mean ± SD; n = 3). Statistical significance was measured by two-tailed Student’s *t* test (*P < .05, †*P* < .01, and ‡*P* < .001). **C**) Treatment conditions of **(B)** were used. Caspase 3/7 activity in treated samples is normalized to untreated control and presented as fold-increase. Results are presented as mean ± SD (n = 3), and statistical analysis was performed as in **(B)**. **D**) Apoptosis was assessed by immunoblotting of PARP cleavage. The SW48 and DLD-1 cell lines were treated with 50 μM or 100 μM oxaliplatin, respectively, in combination with SB202190 (10 μM) or pimasertib (10 μM). Calnexin was used as a loading control. The result presented is representative of three independent experiments. **E**) SW48 and DLD-1 cells were transfected with scrambled or p38 siRNA (50nM and 100nM, respectively). After 48 hours’ siRNA transfection, cells were treated for 24 hours with 50 μM (SW48) or 100 μM (DLD-1) oxaliplatin. Calnexin was used as loading control. The results presented are representative of three independent experiments.

Oxaliplatin treatment induced activation of both p38 and ERK1/2 kinases in SW48 cells (Figure 5A). The combination of oxaliplatin with cetuximab or NAC reduced phosphorylation levels of p38 but not of ERK1/2 (Figure 5A). In contrast, levels of P-p38 and P-ERK1/2 were not affected by SN-38 treatment alone or in combination with cetuximab or NAC (Figure 5A). Activation and nuclear translocation of P-p38 was also detected by immunofluorescence. Nuclear/cytoplasmic ratio of P-p38 was increased by oxaliplatin and reduced by cetuximab (data not shown).

The p38 inhibitor SB202190 and the MEK inhibitor pimasertib were used to determine the roles of p38 MAPK and ERK kinases on apoptosis induced by oxaliplatin in SW48 and DLD-1 cells. The combination of SB202190 with oxaliplatin induced a statistically significant dose-dependent increase in cell viability (oxaliplatin vs oxaliplatin + 100nM SB202190, mean difference = 19.1%, 95% CI = 2.95 to 35.31, *P =* .03; oxaliplatin vs oxaliplatin + 1 μM SB202190, mean difference = 28.9%, 95% CI = 9.19 to 48.54, *P =* .01; oxaliplatin vs oxaliplatin + 10 μM SB202190, mean difference = 34.4%, 95% CI = 16.60 to 52.19, *P =* .05), indicating a role for this pathway in cell survival (Figure 5B). There was a dose-dependent reduction of apoptosis when oxaliplatin was combined with SB202190 (oxaliplatin vs oxaliplatin + 100nM SB202190, mean difference = 3.0-fold, 95% CI = 0.39 to 5.53, *P =* .03; oxaliplatin vs oxaliplatin + 1 μM SB202190, mean difference = 4.0-fold, 95% CI = 1.92 to 6.04, *P =* .005; oxaliplatin vs oxaliplatin + 10 μM SB202190, mean difference = 5.4-fold, 95% CI = 3.70 to 7.12, *P* < .001) (Figure 5C).

In contrast, combination of oxaliplatin with 100nM (oxaliplatin vs oxaliplatin + 100nM pimasertib, mean difference = 20.5%, 95% CI = 3.18 to 37.85, *P =* .03) and 1 μM pimasertib (oxaliplatin vs oxaliplatin + 1 μM pimasertib, mean difference = 18.8%, 95% CI = 3.79 to 33.78, *P =* .02) statistically significantly reduced cell viability (Figure 5B). However, when oxaliplatin was combined with pimasertib (100nM, 1 μM, and 10 μM), cytotoxic potency of oxaliplatin was not affected at any concentrations tested (Figure 5C).

Levels of PARP cleavage produced by oxaliplatin were reduced following treatment with SB202190 and upon p38 knockdown whereas levels of cleaved PARP remained unchanged when oxaliplatin was combined with pimasertib in SW48 and DLD-1 cells (Figure 5, D and E). Activation of Hsp27 and ERK were used to assess SB202190 and pimasertib activity, respectively. SB202190 binds the ATP pocket of p38 and inhibits phosphorylation of downstream proteins but cannot block activation of p38 from upstream kinases (Figure 5D).

## Discussion

Combinations of cetuximab with oxaliplatin in colon cancer have been widely investigated clinically. Some studies have shown benefit from addition of cetuximab with oxaliplatin (25) while others have shown either no benefit (13,26) or a negative interaction (14). In accordance with findings from the COIN, NORDIC, and NEW EPOC studies, we observed negative effects combining cetuximab with oxaliplatin in SW48 and DLD-1 KRAS wild-type cells. Antagonism by platinum drugs and cetuximab on proliferation of KRAS wild-type CRCs has been reported (27). However, positive effects of cetxuimab and oxaliplatin combinations in vitro have also been observed (28–29).

There is evidence for the role of ROS in mediating chemosensitivity (19–21,30). Our results indicate that ROS production is critical for cytotoxicity of oxaliplatin but not of SN-38. Other preclinical studies have shown that treatment with platinum drugs and cetuximab in combination with gefitinib in colon and lung cancer cells resulted in antagonistic effects because of inhibition of chemotherapy-induced ROS by EGFR-targeted agents (27,31). In contrast, others have shown that EGFR inhibition by erlotinib resulted in ROS production via NOX4 overexpression (32).

Our findings indicate a mechanism of ROS production in response to oxaliplatin because of increased levels of the ROS-generating NADPH oxidase enzyme DUOX2. Other studies have shown involvement of different NADPH oxidases (NOX) in producing ROS following chemotherapy (27,33–35).

We showed that STAT1 mediates DUOX2 transcription by direct promoter binding following oxaliplatin treatment whereas cetuximab inhibits STAT1 activation, oxaliplatin-induced DUOX2 upregulation, and ROS generation. Cetuximab can inhibit activation of the EGFR pathway as monotherapy or in combination with chemotherapy (36–38). The inhibitory effect of cetuximab on EGFR and ROS production impairs activation of ROS-dependent cell death mechanisms induced by oxaliplatin via phosphorylation of p38. Activation of mitogen-activated protein kinases (MAPKs), including p38, in response to cisplatin is associated with increased ROS generation and apoptosis (39).

Our in vitro data indicates that DUOX2/DUOXA2 induction by oxaliplatin is a dynamic process occuring within 24 hours. Furthermore, antagonistic effect of cetuximab and oxaliplatin combinations on the apoptosis of SW48 and DLD-1 cells was also observed at this time point, which was therefore used in xenograft experiments. In support of in vitro data, the combination of cetuximab and oxaliplatin showed higher tumor proliferation compared with oxaliplatin alone as measured by Ki67 staining and reduced cleaved caspase 3 levels. DUOX2/DUOXA2 expression was also in accordance with in vitro findings; expression was increased by oxaliplatin and reduced by combination with cetuximab although statistical significance was only reached by DUOXA2.

DUOX enzymes are differentially regulated by immune cytokines (40–42), but it is likely that regulation of DUOX2/DUOXA2 expression in mouse xenografts and human tumors in response to oxaliplatin and cetuximab treatment is more complex. For example, modulation of DUOX2 expression could be by direct effect of drugs on tumors, mouse/human immune response to the tumor cells influenced directly or indirectly by drug exposure, and a response to tumor cell death caused by the drugs.

Our study has limitations. The effects described need to be demonstrated in clinical samples from patients receiving therapy. The effects shown here by combination treatment on ROS production need to be balanced against the contribution of EGFR inhibition on DNA repair and immune responses that would potentially favor this combination (43–44). This might explain why the interaction of EGFR-inhibitory antibodies and chemotherapy is beneficial in some studies (11,12). It is therefore critical to assess the effects of agents used in combination therapy by additional approaches, including patient-derived xenografts and analysis of circulating tumor cells.

However, we have described an important mechanism of negative interaction between oxaliplatin and cetuximab that might explain why a subset of CRC patients does not respond to this combination. Our study also provides a novel explanation for unexpected negative trial results and could be used for optimization of future combination therapies.

## Funding

This work was supported by the Cancer Research UK Programme Grant (C2259/A16569) and by the Medical Research Council for the Council’s Co-operative Awards in Science and Engineering (CASE). HT and AN are Constance Travis postgraduate fellows.

## Supplementary Material

Supplementary Data

## Notes

The study funders had no role in the design of the study; the collection, analysis, or interpretation of the data; the writing of the manuscript; or the decision to submit the manuscript for publication.

We thank Dr. Christopher Stroh for intellectual input and support for this work. We thank Professor Bert Vogelstein, who kindly provided DLD-1 isogenic KRAS wild-type cell lines used in this study. We thank Kairbaan Hodivala-Dilke for advice on xenograft experiment design and to Julie Holdsworth and Bruce Williams for carrying out the experiments.
