# Supplementary material for: Role of Reactive Oxygen Species in the Abrogation of Oxaliplatin Activity by Cetuximab in Colorectal Cancer
Source: J Natl Cancer Inst. 2015 Dec 29;108(6):djv394. doi: 10.1093/jnci/djv394 (PMC4864961; doi:10.1093/jnci/djv394)
Supplement: Supplementary Data [file supp_108_6_djv394__index.html]

Role of Reactive Oxygen Species in the Abrogation of Oxaliplatin Activity by Cetuximab in Colorectal Cancer — Supplementary Data 

# Role of Reactive Oxygen Species in the Abrogation of Oxaliplatin Activity by Cetuximab in Colorectal Cancer

## Supplementary Data

Data files

- Supplementary Data - Supplementary Data
