# Supplementary material for: Role of Reactive Oxygen Species in the Abrogation of Oxaliplatin Activity by Cetuximab in Colorectal Cancer
Source: J Natl Cancer Inst. 2015 Dec 29;108(6):djv394. doi: 10.1093/jnci/djv394 (PMC4864961; doi:10.1093/jnci/djv394)
Supplement: Supplementary Data [file supp_djv394_14_0627R1_Hochhauser_supp_mat_112315.docx]

**Supplementary Figures**

**
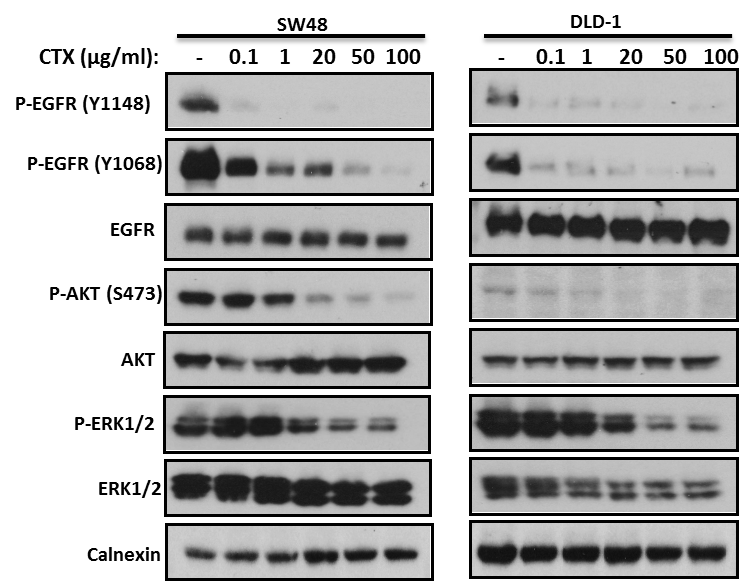
Supplementary Figure 1. Dose-dependent inhibition of the EGFR pathway by cetuximab.** SW48 and DLD-1 cells were treated with cetuximab (0.1-1-20-50-100µg/ml) for 6h. Proteins were extracted and analysed by immunoblotting. Calnexin was used as a loading control. Result presented is representative of three independent experiments.

**A**

**
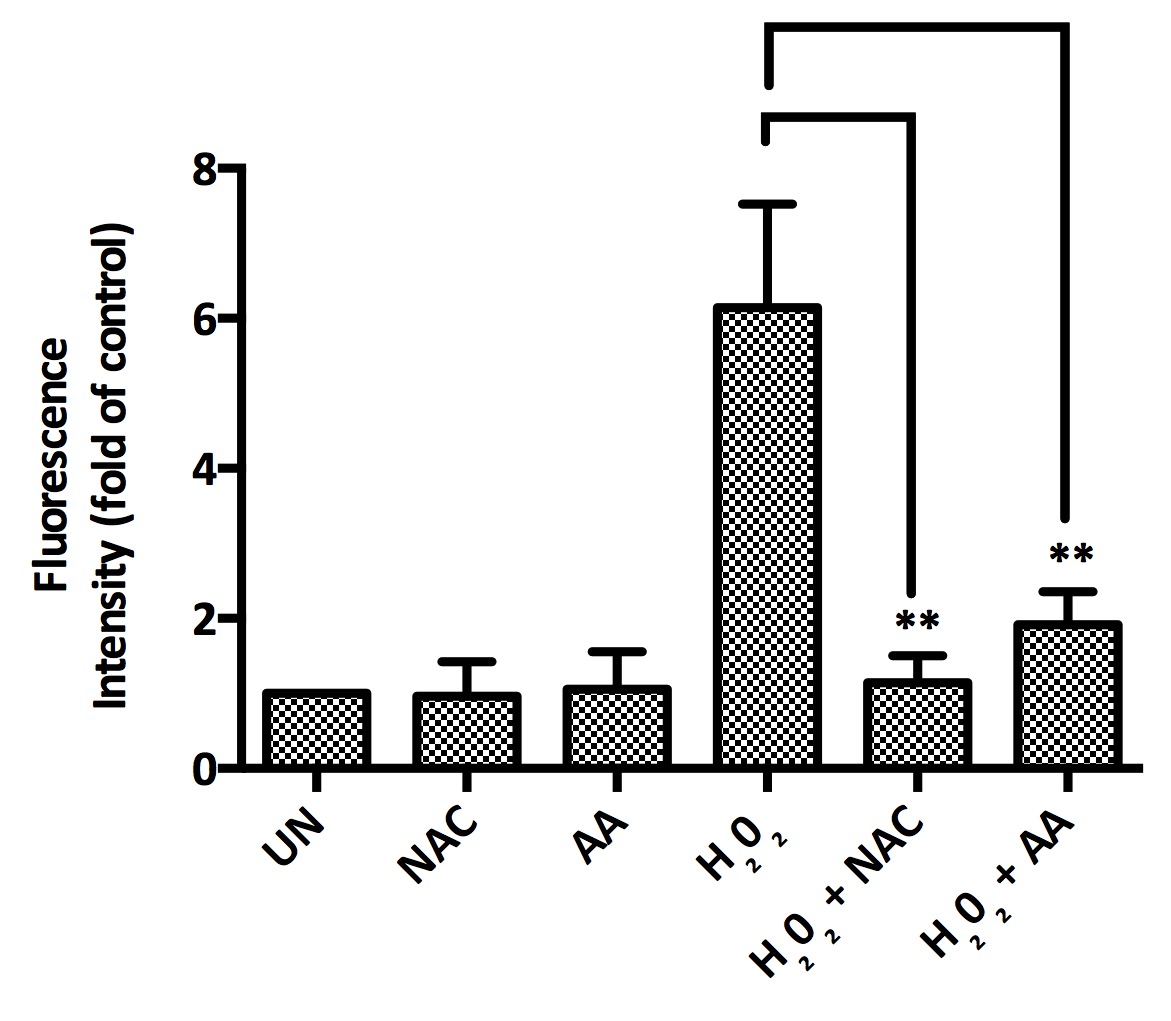
**

**B**


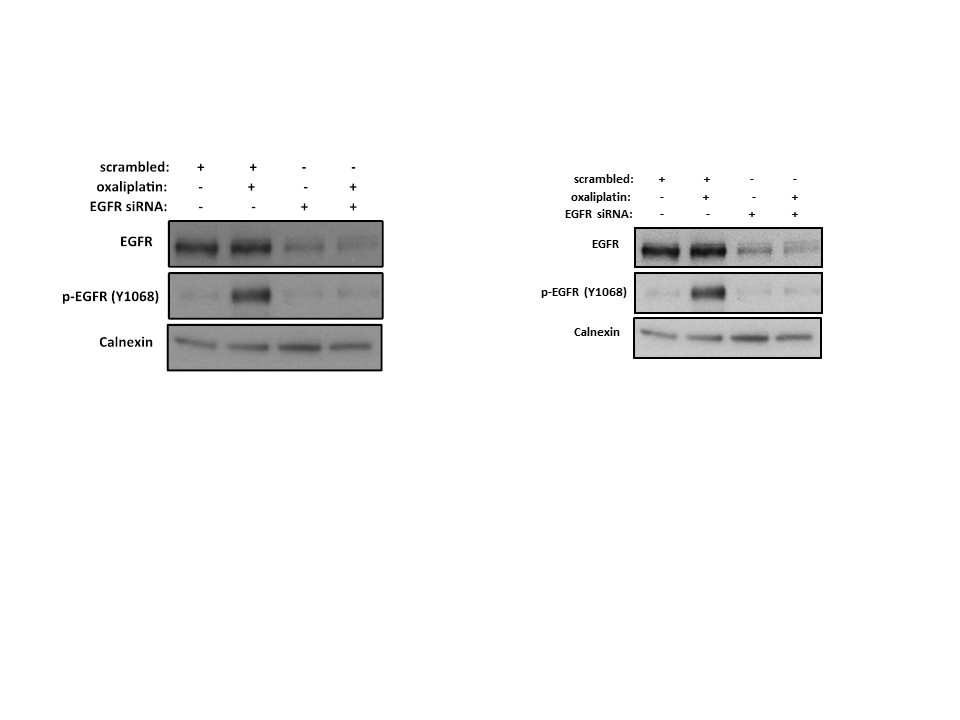


**Supplementary Figure 2. Modulation of ROS levels by oxaliplatin and antioxidant treatments. A**) Levels of ROS were detected with the cell permeable dye DCFDA and FACS analysis. SW48 cells were treated with H_2_0_2_ (1mM) alone or in combination with either NAC (1mM) or ascorbic acid (100μM) for 1h. Results are presented as fold increase to untreated sample and are shown as mean ± SD (n=3). Statistical analysis was performed with two-tailed Student’s *t* test (*P< . 05, **P< .01, ***P< .001). **B**) SW48 cells were transfected for 72h with scrambled siRNA or EGFR siRNA (50nM) and subsequently treated with oxaliplatin (50μM), cetuximab (100μg/mL) or their combination for 1h. Calnexin was used as loading control. In the same experimental conditions ROS levels were measured. Experiment was repeated three times and result is shown as mean ± SD. Statistical analysis was performed with two-tailed Student’s *t* test (*P<. 05, **P<. 01, ***P< .001).

**Supplementary Figure 3. Dose and time-dependent effect of gefitinib on the EGFR pathway.** SW48 and DLD-1 cells were treated with gefitinib (1 and 5μM) for 6h. Proteins were extracted and analysed by Immunoblotting with the indicated antibodies. Calnexin was used as a loading control. Result presented is representative of three independent experiments.

**A**

**B**


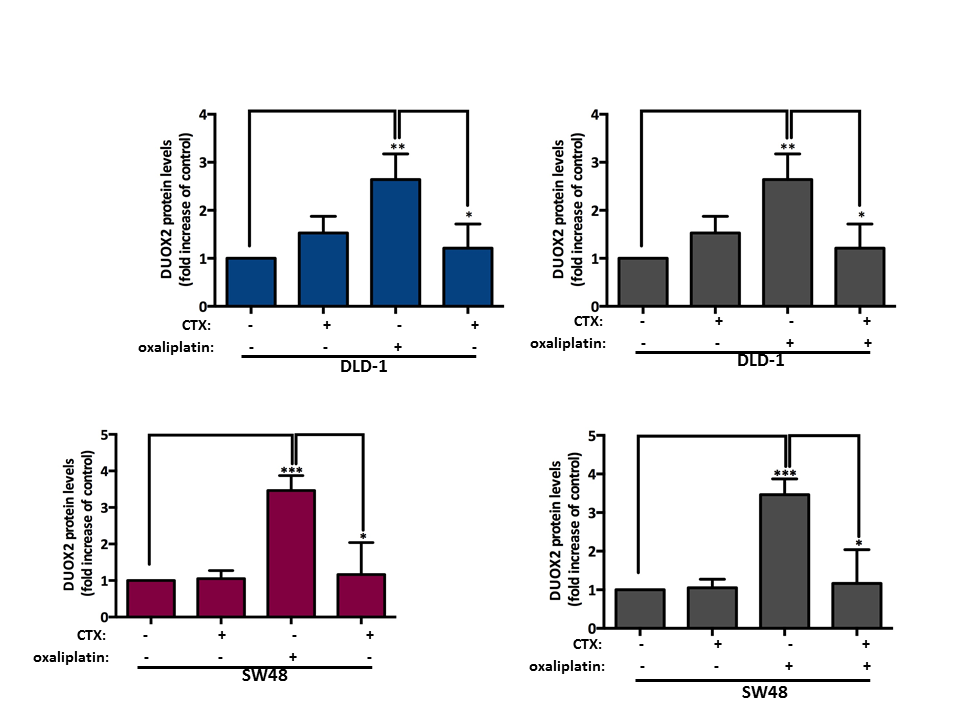

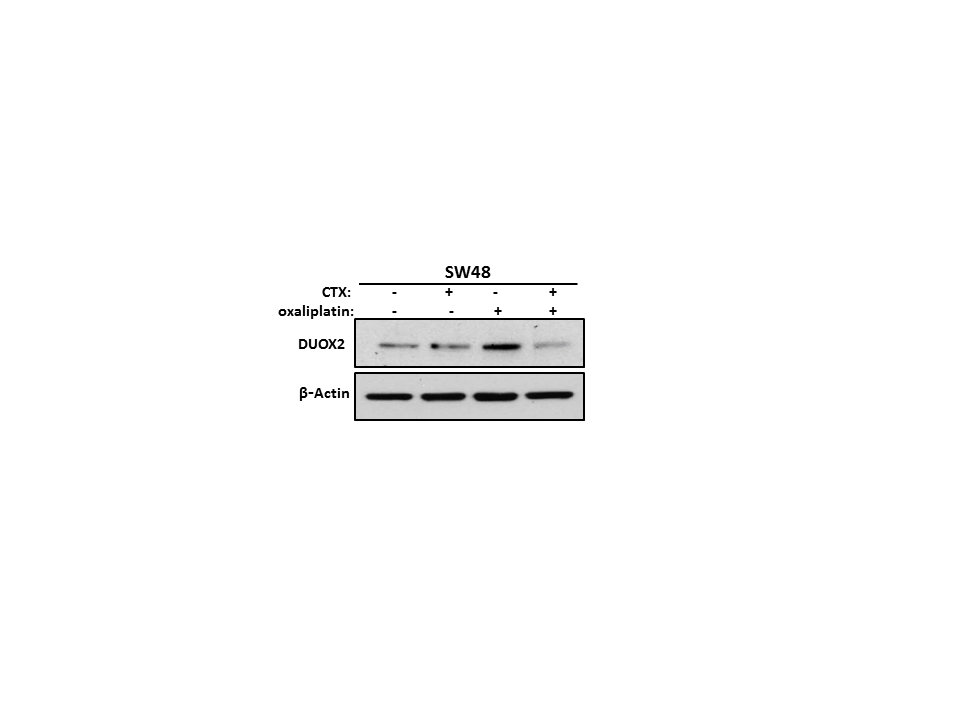


**C**


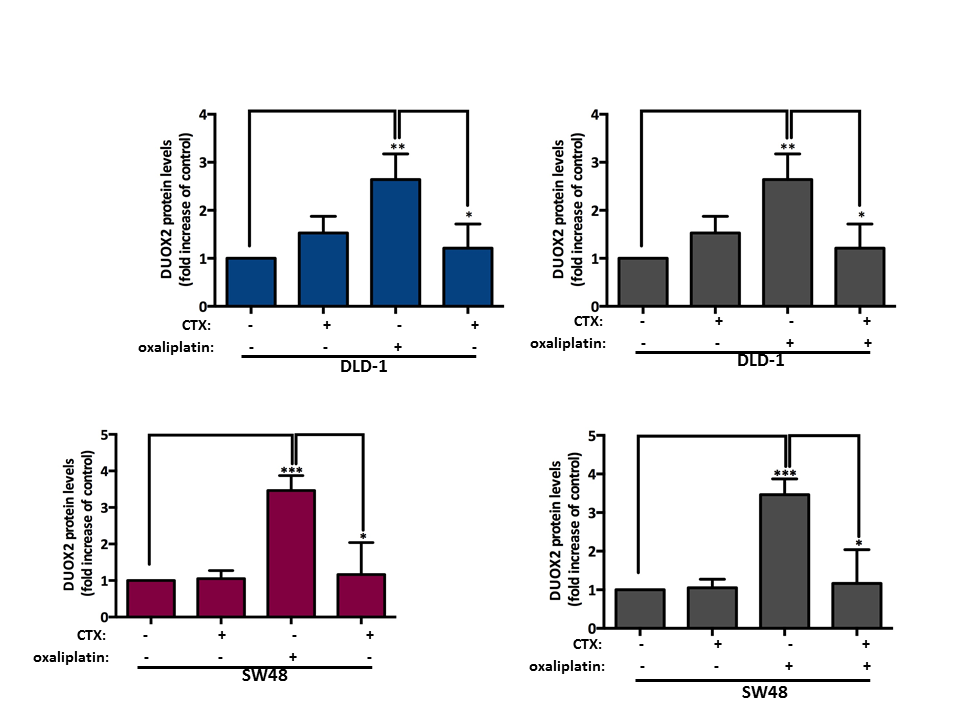

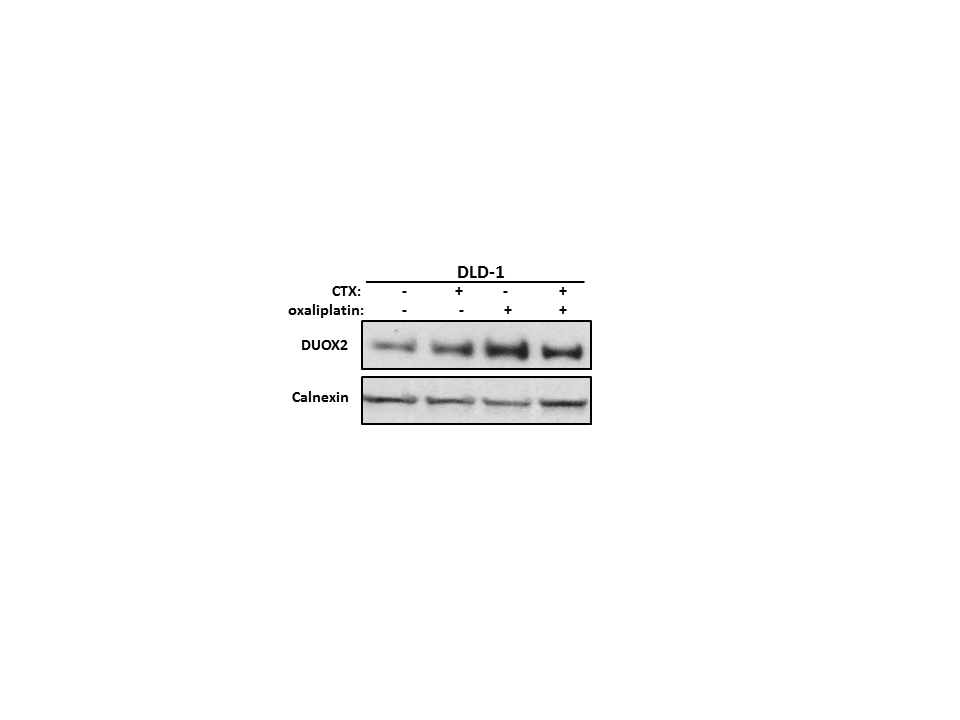

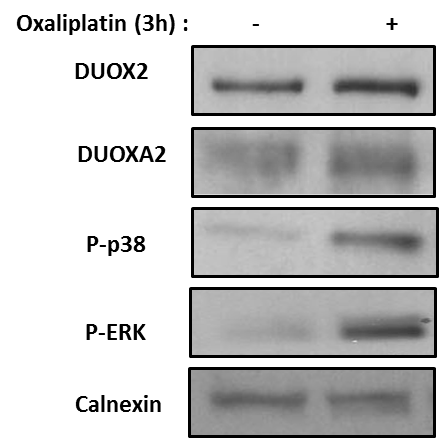


**Oxaliplatin: - +**

**D**

**
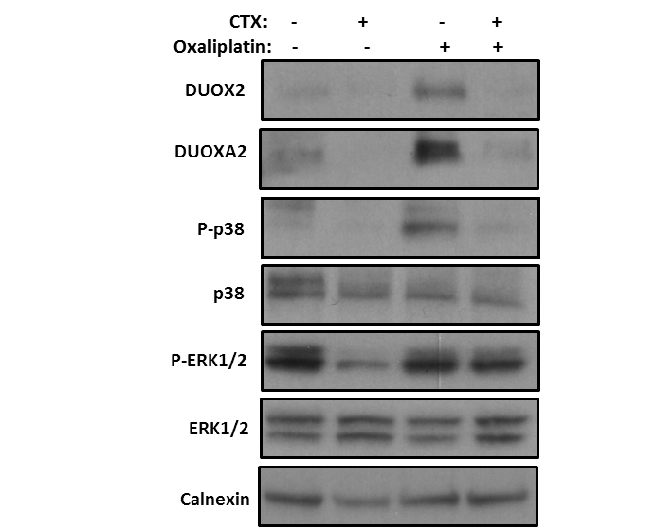
**

**E**


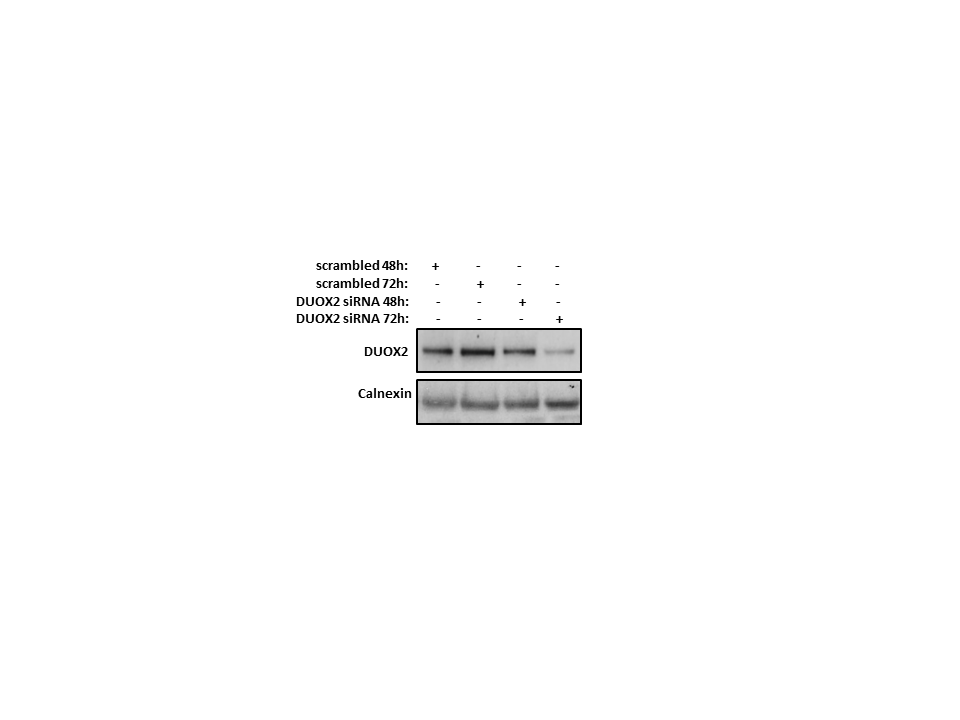


**Supplementary Figure 4. Oxaliplatin and cetuximab modulate DUOX2/DUOXA2 expression and ROS generation in SW48 and DLD-1 cells. A)** SW48 cells were treated with oxaliplatin (50μM), cetuximab (100μg/mL) or their combination for 6h. In **B**) DLD-1 cells were treated with oxaliplatin (100μM), cetuximab (100μg/mL) or their combination for 6h. Protein samples were collected and processed by Immunblotting. β-Actin was used as a loading control (left panel). Densitometry analysis (Image J software) of three independent experiments was conducted. Results are presented as mean ± SD and statistical significance was calculated by two-tailed Student’s *t* test (right panel) (*P< .05, **P< .01, ***P< .001). DLD-1 cells (**C**) and SW48 cells (**D**) were treated as in A and B. Samples processed by immunoblotting and membranes incubated with the indicated antibodies. Calnexin was used as a loading control. Result is representative of three experiments. **E**) SW48 cells were treated with DUOX2 siRNA and control siRNA (50nM) for the indicated times. Calnexin was used as a loading control (left panel). Following 72h siRNA transfection, SW48 cells were treated for 6h with oxaliplatin (50μM). ROS levels were measured by flow cytometry and plotted as mean fluorescence intensity normalized to untreated control. Results are presented as mean ± SD and statistical significance was calculated by two-tailed Student’s *t* test (right panel) (*P<. 05, **P< .01, ***P< .001).

**
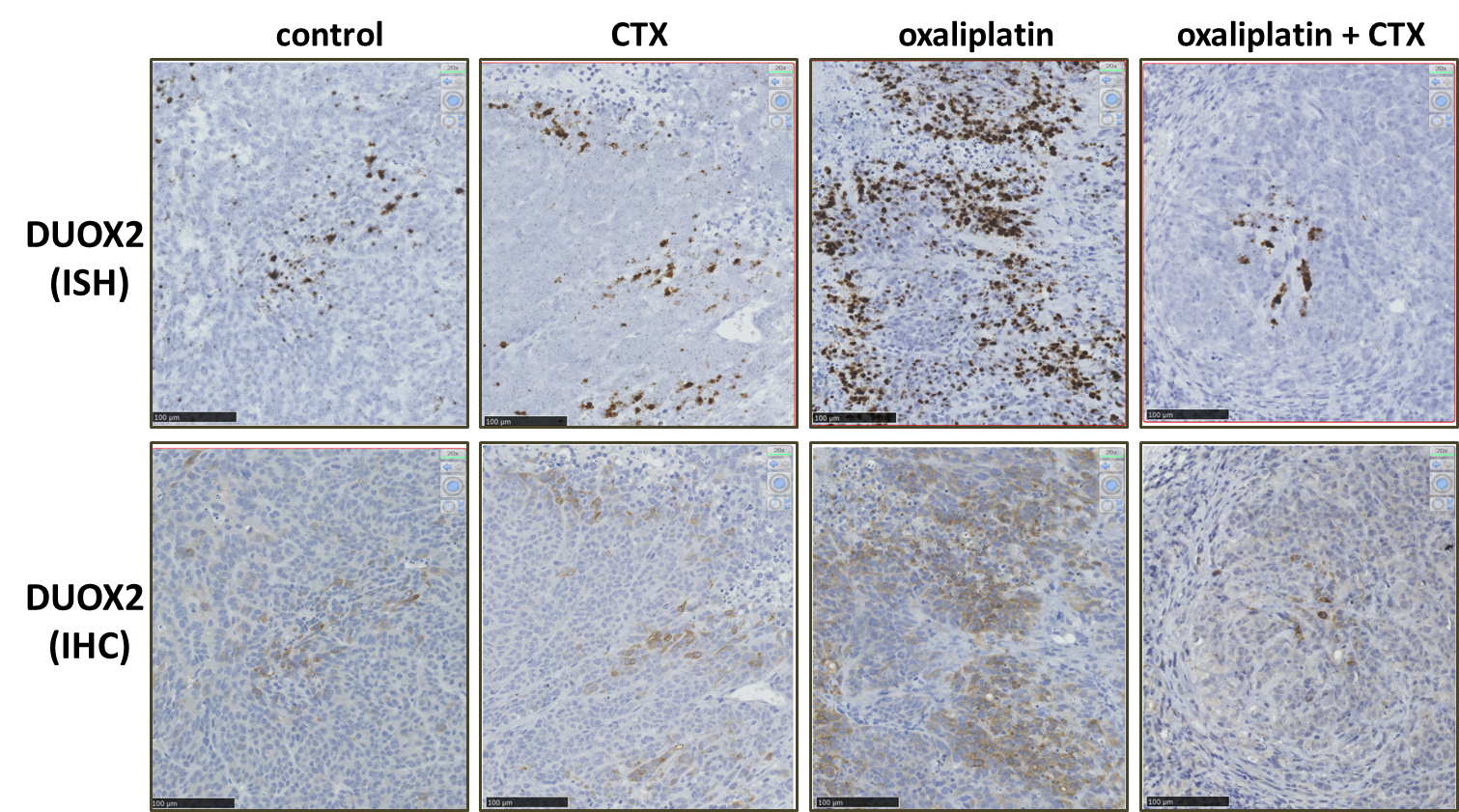
**

**Supplementary Figure 5. DUOX2 expression following oxaliplatin and cetuximab treatment was measured in DLD-1 xenografts.** DLD-1 Xenograft sections (5μM) were prepared and DUOX2 *in situ* hybridisation (ISH) and DUOX2 immunohistochemistry (IHC) conducted. Number of mice used were control and n=3 per treatment group. Representative images at 20x magnification resulting from ISH and IHC analysis are presented. Scale bars = 100µM.

**A**

**
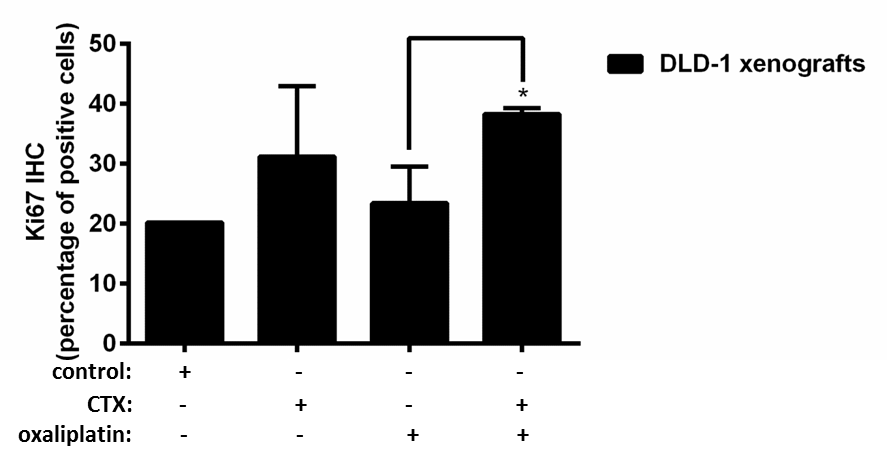
**

**B**


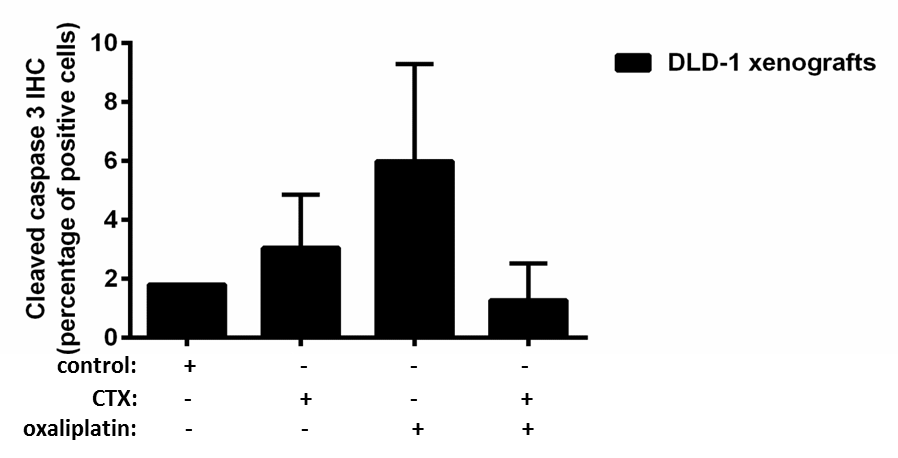
**Supplementary Figure 6. Effects of oxaliplatin and cetuximab on proliferation and apoptosis *in vivo*.** Proliferation and apoptosis of DLD-1 xenografts treated with cetuximab, oxaliplatin or combination were assessed using Ki67 (**A**) and cleaved caspase 3 (**B**) with results expressed as percentage positive cells (mean ± SD). Number of mice used were control and n=3 per treatment group. Statistics was performed using two-tailed, Student’s *t*- test with Welch’s correction (*P< .05, **P< .01, ***P< .001).

**Supplementary Materials and Methods**

**Reagents and antibodies**

The following antibodies were purchased by Cell Signaling Technology: EGFR (rabbit monoclonal, 1:1000), p-EGFR (Tyr-1068) (rabbit monoclonal, 1:1000), Extracellular Signal Regulated Kinase (ERK1/2) (rabbit monoclonal, 1:1000), pERK1/2 (Thr202/Tyr204) (rabbit monoclonal, 1:1000), P-p38α MAPK (Thr180/Tyr182) (rabbit monoclonal, 1:1000), p38α MAPK (rabbit monoclonal, 1:1000), p-MAPKAP2 (rabbit monoclonal, 1:1000), Cleaved PARP (rabbit monoclonal, 1:1000), β-Actin (rabbit monoclonal, 1:5000), Calnexin (rabbit monoclonal, 1:1000), p-HSP27 (Ser82) (rabbit monoclonal, 1:1000), p-STAT1 (Ser727) (rabbit monoclonal, 1:1000), STAT1 and Normal Rabbit IgG Ab. STAT1 Ab (ChIP specific) (rabbit polyclonal, 1:50) and Dual oxidase 2 maturation factor (DUOXA2) Ab were purchased from Santa-Cruz Biotechnology (rabbit polyclonal, 1:500), p-Histone H2A.X from Millipore (mouse monoclonal, 1:1000), DUOX2 Ab from Abcam (rabbit monoclonal, 1:1000) and the secondary fluorescent conjugated goat anti-rabbit antibody Alexa Fluor 488 from Life Technologies (rabbit monoclonal, 1:500). Smart-pool siRNA for human EGFR, STAT1, DUOX2 and non-targeting controls were obtained from Thermo-Scientific. ROS detection reagent H_2_DCFDA was purchased from Life Technologies.

**Immunoblotting**

Protein extracts were obtained with CelLytic™ M cell lysis reagent (Sigma-Aldrich) and denatured by heating for 5 min at 95° in sample buffer containing 100 mM Tris-Cl (pH 6.8), 4% SDS, 10% 2-mercaptoethanol, 20% glycerol, and 0.02% bromophenol blue (Life Technologies) and resolved on a 7% Tris-acetate, 10% or 4-12% Bis-Tris NuPAGE gel (Novex, Pre-cast gels, Life Technologies). 10X Running NuPAGE [60.5g Tris-Base (Sigma-Aldrich), 89.5g Tricine (Sigma-Aldrich) and 10g of SDS (Sigma-Aldrich)] or MOPS [52.3g MOPS (Sigma-Aldrich), 30.3g Tris-Base (Sigma-Aldrich), 5g SDS (Sigma-Aldrich) and 1.5g EDTA (Sigma-Aldrich)] running buffers were diluted to 1X with distilled water and were used for protein separation. Proteins were subsequently transferred to polyvinylidene difluoride membranes (Immobilon®-P transfer membrane; Millipore) activated by immersion in MeOH (VWR) for 30sec. The transfer buffer was prepared with a 1:10 dilution of 10X Tris-Glycine buffer [30.3g Tris-Base (Sigma-Aldrich) and 144.1g Glycine (Sigma-Aldrich)] in distilled water and 20% Methanol. Proteins were transferred at 35V for 2-2.5h at room temperature using the XCell II Blot module (Life Technologies) and membranes were blocked for 1h at room temperature in blocking buffer containing 5% BSA (Sigma-Aldrich) in 1x TBS, 0.1% Tween-20 (Sigma-Aldrich). All primary antibodies were incubated overnight at 4°. Anti-rabbit or mouse IgG, HRP-linked Antibody (Cell Signaling Technologies) were used to detect primary antibody. Enhanced chemiluminescence (ECL system, Amersham) was used to detect proteins of interest.

**Drug combination assays**

Cells were treated with IC_20_ dose cetuximab (1.4μg/mL) and/or increasing concentrations of oxaliplatin (1-2-3-5μM) or SN-38 (0.01-0.1-1μM) for 72h (non-fixed ratio drug combination). Following drug treatments cells were incubated with 20μL/well MTT (5mg/mL) for 4h at 37°. Formazan crystals were solubilised in 200μL DMSO and absorbance was measured at 540nm with the Varioskan Plate reader. The Calcusyn software (Biosoft) was used for calculation of combination indices according to non-fixed ratio design using methodology of Chou and Talalay ([23](#_ENREF_23)). A median effect plot using linear regression of dose-response data was used to calculate a combination index value that describes the interaction between the two drugs at each point studied. Combination indices of 1 indicate additive effects, when > 1 antagonistic effects and when <1 synergistic effects.

**Chromatin Immunoprecipitation (ChIP)**

SW48 cells treated with oxaliplatin were lysed on ice with Lysis Buffer [5mM PIPES pH (8.0), 85mM KCL, 0.5% NP40] for 15min and centrifuged at 2500rpm for 5min at 4°. Pellets were resuspended in Nuclear Lysis Buffer (50mM Tris-HCL pH 8.0, 10mM EDTA and 0.1% SDS). Chromatin was sonicated (50% amplitude) on ice with 6 pulses of 45sec followed by 30sec intervals. Pre-cleared [2h at 4° with Protein A beads (GE Healthcare)] chromatin samples were incubated with 8μg of STAT1 Ab (sc-346 X, Santa Cruz) or 8μg of Normal Rabbit IgG (Cell Signaling) at 4° overnight. Blocked Protein A beads [BSA 1μg/μL (Sigma-Aldrich) and Salmon Sperm DNA 1μg/μL (Life Technologies)] were used to immunoprecipitate (IP) STAT1-DNA complex. IP samples and INPUT samples were reverse-crosslinked with 10μg RNAse (Thermo Scientific) at 65° overnight. DNA was extracted and used as template for RT-PCR amplification [2X SYBER Green Master Mix (Qiagen)] of a specific STAT1 binding site (232 bp region) on the DUOX2 promoter. The following primers were used: forward 5’-AAACCAGAGTCCCCAAGACC-3’ and reverse 5’-GGAGTGAAGGTGGTGGAAGA-3’. RT-PCR protocol consisted of an initial incubation at 95°C for 15 minutes followed by 40 cycles at 95°C (15 seconds), 1 minute annealing at 58°C, 1min extension at 72°C and complete primer dissociation curve.

**RT-PCR Oxidative stress arrays**

Total RNA was extracted from cells treated with 50μM oxaliplatin, 100μg/mL cetuximab or combination for 6h using the RNeasy kit (Qiagen) according to manufacturer’s protocol. Template cDNA was generated using RT^2^ First Strand Kit (SABiosciences, Qiagen), amplified using RT^2^ qPCR Mastermix (SABiosciences-Qiagen) and PCR performed according to the manufacturer’s protocol. Gene expression analysis was conducted using SABiosciences online tool (<http://www.sabiosciences.com/pcr/arrayanalysis.php>).

**Real-time PCR of DLD-1 xenografts tissue samples**

mRNA for DUOX2 and DUOXA2 was determined as follows. Xenograft tissue was harvested and placed immediately into AllProtect solution (Qiagen, UK). This was stored at 4°C for a maximum of 24 hours before removal for RNA extraction. Total RNA was extracted using the miRNeasy kit (Qiagen, UK) according to manufacturer’s protocol. RNA concentration was measured using the NanoDrop Spectometre (Nano-Drop Technologies, USA). cDNA was obtained by using the High-Capacity-RNA to cDNA kit (Applied Biosystems, UK) in a 20 μl reaction. cDNA was then incubated with Taqman assays (20x) and Universal Mastermix no UNG (Applied Biosystems, UK) on a 7500 Fast System RealTime PCR cycler according to manufacturer’s protocol. Human GAPDH was used as the housekeeping gene. Fold changes were calculated using the 2^-ΔΔCt method.

**In situ hybridisation and immunohistochemistry**

Xenograft tissue was harvested and immediately placed in neutral buffered formalin (10%) (Sigma, UK) for 12 hours. Tissue was then placed in ethanol (70%) for no longer than 24 hour before paraffin embedding. Serial sections (5 µm) were cut for In situ hybridization (ISH) and immunohistochemistry (IHC).

For ISH, hDUOX2 expression was determined using the RNAscope 2.0 High Definition (Brown, catalog number 310035) assay according to the manufacturer’s instructions (Advanced Cell Diagnostics, Hayward, CA). Briefly, samples were heated at 60**˚**C for 1 hour, followed by dewaxing and rehydrating in water and incubation with Pretreatment 1 buffer for 10 minutes at room temperature (RT). Slides were boiled in Pretreatment 2 buffer for 15 minutes, followed by incubation with Pretreatment 3 buffer for 30 minutes at 40˚C. Slides were incubated with the probe for 2 hours at 40**˚**C, followed by successive incubations with Amp1 to 6 reagents. Staining was visualized with 3,3-diaminobenzine (DAB) then lightly counterstained with haematoxylin before dehydrating and mounting with DPX.

IHC was performed according to standard protocols. Briefly, serial sections were dewaxed, rehydrated and immersed in 3% hydrogen peroxide for 10 minutes to quench endogenous peroxidase activity. Antigen retrieval was performed by microwaving for 15 minutes in sodium citrate buffer (pH6.0). After cooling, sections were incubated with blocking buffer (1:25 goat serum in PBS) for 15 mins at RT. Primary antibodies were applied for 45 minutes at RT (rabbit anti-human Ki67 [ab92742] at 1:1000 dilution, Abcam plc, Cambridge, UK; or rabbit anti-human cleaved caspase 3 [cell signaling 9664] at 1:400). Sections were then incubated with a biotinylated secondary antibody at RT for 45 min, followed by incubation with streptavidin-biotin peroxidase solution at RT for 45 min. Visualization of antibody binding was carried out using DAB and sections were lightly counterstained using haematoxylin as before.

Slides were scanned using a NanoZoomer 2.0 H-T, (Hamamatsu Photonics UK Limited, Welwyn Garden City, UK) and saved as ndpi files. Images were viewed using NDPview2 for Macintosh Files and each target under investigation was aligned across serial sections. Seven randomly selected areas were then examined at x20 or x40 magnification. Total cell numbers and the number of positively stained cells within a field were counted for IHC and expressed as percentage positive cells. For ISH a positive cell was scored if a minimum of 3 dots could be seen within the cell boundary.

**References**

23. Chou TC. Drug combination studies and their synergy quantification using the Chou-Talalay method. *Cancer Res*. 2010;70(2):440-6.
